# Protective effect of ischaemic postconditioning combined with nicorandil on myocardial ischaemia‒reperfusion injury in diabetic rats

**DOI:** 10.1186/s12872-022-02967-1

**Published:** 2022-12-03

**Authors:** Zongyi Xia, Bing Chen, Chi Zhou, Yitian Wang, Jinyang Ren, Xujin Yao, Yifan Yang, Qi Wan, Zhexun Lian

**Affiliations:** 1 Department of Cardiology, The Affiliated Hospital of Qingdao University, 16 Jiangsu Road, Qingdao, 266003 Shandong China; 2 Institute of Neuroregeneration & Neurorehabilitation, Department of Pathophysiology, Qingdao University, 308 Ningxia Street, Qingdao, 266071 Shandong China; 3 Department of Genetics and Cell Biology, Basic Medical College, Qingdao University, 308 Ningxia Street, Qingdao, 266071 Shandong China

**Keywords:** Ischaemia postconditioning, Myocardial ischaemia‒reperfusion injury, Nicorandil, PI3K/Akt signalling pathway, Type 2 diabetes mellitus

## Abstract

**Background:**

The diabetic heart exhibits a high sensitivity to ischaemia/reperfusion (I/R) injury. Diabetes mellitus (DM) can affect the efficacy of cardioprotective interventions and reduce the therapeutic potential of existing treatment options. This study aimed to investigate the feasibility of shifting from monotherapy to combination therapy in diabetic myocardial I/R injury.

**Methods:**

6–8 week rats were randomized into 10 groups: sham, I/R, ischaemia postconditioning (I-Post), nicorandil (Nic), combination therapy (I-Post + Nic), DM sham, DM I/R, DM I-Post, DM Nic and DM I-Post + Nic. The extent of myocardial injury was clarified by measuring CK-MB and NO levels in plasma, ROS content in myocardial tissues, and TTC/Evans Blue staining to assess the area of myocardial infarction. Pathological staining of cardiac tissue sections were performed to clarify the structural changes in myocardial histopathology. Finally, Western blotting was performed to detect the phosphorylation levels of some key proteins in the PI3K/Akt signalling pathway in myocardial tissues.

**Results:**

We confirms that myocardial injury in diabetic I/R rats remained at a high level after treatment with I-Post or nicorandil alone. I-Post combined with nicorandil showed better therapeutic effects in diabetic I/R rats, and the combined treatment further reduced the area of myocardial injury in diabetic I/R rats compared with I-Post or nicorandil treatment alone (*P* < 0.001), as well as the levels of the myocardial injury markers CK-MB and ROS (*P* < 0.001); it also significantly increased plasma NO levels. Pathological staining also showed that diabetic rats benefited significantly from the combination therapy. Further mechanistic studies confirmed this finding. The protein phosphorylation levels of PI3K/Akt signalling pathway in the heart tissue of diabetic I/R rats were significantly higher after the combination treatment than after one treatment alone (all *P* < 0.05).

**Conclusion:**

I-Post combined with nicorandil treatment maintains effective cardioprotection against diabetic myocardial I/R injury by activating the PI3K/Akt signalling pathway.

**Supplementary Information:**

The online version contains supplementary material available at 10.1186/s12872-022-02967-1.

## Introduction

Diabetes is an independent risk factor for cardiovascular disease (CVD), and acute myocardial infarction (AMI) is the leading cause of morbidity and mortality in diabetic patients [1, 2]. Diabetic patients have significantly higher mortality from AMI than nondiabetic patients and tend to develop more severe myocardial ischaemia‒reperfusion injury (myocardial I/R injury) after reperfusion therapy [3], with long-term (≥ 1 year) mortality being nearly 50% higher than that in nondiabetic patients [4]. Therefore, reducing myocardial I/R injury in diabetic patients, preserving the maximum amount of ischaemic myocardium and improving patient prognosis have become urgent challenges for reperfusion therapy.

Ischaemic preconditioning (IPC) is a measure that protects the myocardium from subsequent prolonged ischaemia by subjecting it to brief repeated ischaemia. Subsequently, several studies have shown that this protection against myocardial I/R injury can be induced by intervening in reperfusion [5]. The area of damage caused by myocardial I/R injury can be limited by three sets of very short ischaemia (30 s)-reperfusion (30 s) cycles before reperfusion, i.e., ischaemic postconditioning (I-Post) [6]. Similar to IPC, I-Post activates several signalling pathways related to cardioprotection to protect the myocardium from myocardial I/R injury [7]. Moreover, these signalling pathways can be induced by other interventions or by the administration of drugs and other chemical agents that can attenuate the damage caused by reperfusion by administering drugs at different times of intervention (during or after reperfusion), which is known as pharmacological modulation (PC) [8]. In contrast to IPC, I-Post and PC shift the timing of mechanical or pharmacological intervention from preischaemia (which cannot be performed in most clinical settings) to prereperfusion. These conditions are similar to clinical procedures for the onset and treatment of AMI and have more immediate clinical applications. However, the modulatory role of postconditioning in diabetes has not been thoroughly investigated. Some studies suggested that the cardioprotective effect of I-Post was diminished in both type 1 and type 2 diabetic animal models, while others concluded that I-Post was ineffective in both type 1 and type 2 diabetic rat models [9]. Moreover, in the nearly 20 years since I-Post was discovered, the results of animal experiments have not been translated into clinical applications, which may be explained by the more complex clinical conditions of patients, where age and concomitant diseases such as hypertension, diabetes mellitus, and hypercholesterolemia may affect the performance of protective measures [5]. Therefore, I-Post may have a limited effect on diabetic patients. Furthermore, previous studies have also shown that diabetes impairs the cardioprotective effects of PCs, such as sevoflurane [10] and remifentanil [11]. Undoubtedly, the severity and complexity of diabetic myocardial I/R injury affects the effectiveness of available therapeutic measures.

As mentioned previously, the I-Post intervention is more in accordance with clinical procedures for acute myocardial infarction onset and treatment but limits patient benefit due to comorbidities such as diabetes mellitus. Therefore, the question of how to improve postadaptation to make it more compatible with the actual needs of patients is an urgent issue to be addressed. A study has noted that the way to overcome the ineffectiveness of conditioning interventions in diabetic patients is to provide adjuvant or combination therapy (e.g., antioxidants, antidiabetic drugs) in addition to the conditioning strategy [12]. Therefore, we suggest that the combination of drugs known to be cardioprotective and antidiabetic in addition to I-Post may be a new strategy to address myocardial I/R injury in diabetes. We hypothesized that the I-Post combination PC would be superior to any of the individual interventions and that these interventions would have a superimposed effect when the mechanism of action of any of the interventions is not identical. Combination therapies can be protective even when diabetes may reduce the protective effect of one therapy. One such drug, nicorandil, is widely used to treat ischaemic heart disease (IHD). Several animal studies have shown that nicorandil attenuates myocardial I/R injury [13] and attenuates diabetic cardiomyopathy rat apoptosis in cardiomyocytes [14]. There is still a lack of studies on whether nicorandil can exhibit a protective effect on myocardial I/R injury in diabetic rats. Therefore, we will first investigate the therapeutic effects of I-Post and nicorandil alone on diabetic myocardial I/R injury. Then, we will investigate whether the combined treatment has a better cardioprotective effect on myocardial I/R injury in diabetic rats. In addition, previous studies on diabetic myocardial I/R injury have mostly used the type 1 diabetes model, whereas in actual clinical practice, approximately 90% of diabetic patients have type 2 diabetes mellitus (T2DM) [15]. Therefore, in this study, we used a high-fat and high-sugar diet supplemented with a low-dose streptozotocin (STZ)-induced myocardial I/R model in rats with T2DM. The study aim was to investigate whether I-Post combined with nicorandil exhibits better cardioprotective effects on myocardial I/R injury in T2DM rats and to provide a basis for the clinical treatment of myocardial I/R injury in diabetic patients.

## Materials and methods

### Animals

In this study, we used 150 of 6–8 week old male Sprague–Dawley rats (200-250 g) were used for the experiments, purchased from SPF (Beijing) Biotechnology Co., Ltd. [License No.: SCXK (Beijing) 2019–0010]. The rats were housed in a temperature-controlled environment (21 ± 2 °C) with a 12-h light/dark cycle (lights on at 06:00) and free access to food and water. The facilities where the animals were housed followed the Association for Assessment and Accreditation of Laboratory Animal Care International (AAALAC) guidelines, which were approved at the time of the study. The study protocol was approved by the Ethics Committee of Qingdao University School of Medicine (Qingdao, China).

### Type 2 diabetic rat model

The T2DM model was induced as follows: Experimental animals were fed basal chow for 1 week. After 1 week, the diabetic group was fed high-fat and high-sugar chow, and the control group was fed basal chow. At week 5, all animals were fasted for 12 h (without water), and STZ (1%) was dissolved in 0.1 mmol/L citric acid-sodium citrate buffer (pH 4.2; Solarbio, Beijing, China), fully dissolved and stored on ice away from light. A single rapid intraperitoneal injection of 30 mg/kg streptozotocin (MCE, USA) was administered to the diabetic group. An equal volume of 0.1 mM citric acid-sodium citrate buffer was intraperitoneally administered to the control group. Seven days later, blood was drawn from the tail vein to check for glucose levels of the rats in the diabetic group. Rats with blood glucose ≥ 16.7 mmol/L were fed a high-fat, high-sugar diet for another 8 weeks, while rats that did not meet the blood glucose standard were discarded. Blood glucose was retested after 8 weeks, and modeling was successful if blood glucose was still ≥ 16.7 mmol/L [16].

### Oral glucose tolerance test (OGTT) and insulin tolerance test (ITT)

OGTT: Rats in the normal group (NG: conventional rat chow feeding group) and the diabetes mellitus group (DMG: high-fat chow feeding group supplemented with low-dose STZ injection group) were fasted overnight 12 h before the test, while ensuring access to drinking water. On the day of the test, a 50% glucose solution was prepared, and the volume of glucose solution required for each rat to be tested was calculated based on body weight (2 g glucose/kg body weight) after weighing the rats to be tested. After the gavage operation, blood was taken through the tail vein to test the blood glucose of rats at 0 min, 30 min, 60 min, 90 min and 120 min, and a line graph was made and the area under the curve (AUC) was calculated to compare the difference in blood glucose between the two groups. ITT: Two groups of rats, NG and DMG, were taken and fasted 5 h before the test, while ensuring access to drinking water. And the insulin aspart was diluted in 0.9% saline to prepare a 1:1000 solution (NovoRapid: 100 U/mL; working concentration 0.1 U/mL). After fasting, the rats to be tested were weighed and the volume of solution required for each rat to be tested was calculated based on body weight (0.5 U of menadione insulin/kg body weight). After intraperitoneal injection of insulin, blood was collected through the tail vein to test the blood glucose of rats at 0 min, 30 min, 60 min, 90 min and 120 min, and a line graph was made and the area under the curve was calculated to compare the difference in blood glucose between the two groups.

### Myocardial ischaemia‒reperfusion model

The rats were weighed, fasted, and dehydrated for 12 h before surgery. Sodium pentobarbital (50 mg/kg) was injected intraperitoneally (Item No. P3761, Sigma, USA). Each rat was fixed in the supine position and connected to a Powerlab data acquisition and analysis system. A standard II-lead ECG was recorded, and any abnormalities were excluded. The cervical trachea was incised, and a small animal ventilator (Rivard, USA) was connected for assisted breathing (tidal volume, 5 mL/100 g, frequency 60–80 breaths/min, respiratory ratio 2:1, continuous positive end-expiratory pressure). The skin was cut longitudinally 0.5 cm on the left side of the sternum, using the 3rd and 4th ribs as the upper and lower borders. The subcutaneous tissue, pectoralis major muscle, and intercostal muscle were bluntly separated with forceps and a scalpel. Then, 6–0 ophthalmic sutures were passed under the left atrium and 2–3 mm below the intersection of the cone of the pulmonary artery. The left anterior descending branch of the coronary artery was ligated for 30 min, after which the sutures were cut. After successful ligation, the anterior wall of the left ventricle was bruised or pale, the pulsation was reduced, and the ECG showed ST-segment elevation (≥ 0.25 mV), which is a sign of myocardial ischemia. Thirty minutes after ligation, the ligature was cut with scissors to form a reperfusion, and the ECG showed a gradual decrease in the ST segment by approximately 50% and the pale or cyanotic myocardium gradually turned red when blood flow was restored. Reperfusion was allowed to occur for 2.5 h [17]. After the end of reperfusion, rats were euthanized by a single intraperitoneal injection of an overdose (150 mg/kg) of sodium pentobarbital (Item No. P3761, Sigma, USA).

### Nicorandil administration route and dosage

In this study, Nicorandil for injection (trade name: Ricoxyl; specification: 12 mg; State Drug Administration H20120069) was used, and the dried drug powder was prepared into a solution of 100 μg/ml with 0.9% saline before the experiment and stored away from light. We selected the effective dose in the clinical study, a loading dose of 200 μg/kg of nicorandil was given via femoral vein 20 min before reperfusion, and a maintenance dose of 20 μg/kg/min was given for 60 min after reperfusion [18].

### Experimental groups

A total of 10 groups were included in this study, with 15 animals in each group. (Flowchart of experimental protocol is shown in the Additional file 1: Figure S1).

1. Sham group: The sutures were threaded, but the descending artery was not ligated. The rats were sacrificed 3 h later.
2. I/R group: Rats were treated as described above.
3. I-Post group: After ligation, the rats were treated for myocardial ischemia (30 s ischemia/30 s reperfusion given 3 times within 3 min of the start of reperfusion). Then, reperfusion was allowed to occur for 2.5 h.
4. Nic group: A loading dose of nicorandil (200 μg/kg) was administered 20 min before reperfusion. Then, a maintenance dose (20 μg/kg/min) was administered for 60 min during reperfusion. Afterwards, reperfusion was permitted for another 1.5 h (2.5 h total reperfusion time).
5. I-Post + Nic group: Rats were administered a loading dose of 200 μg/kg of nicorandil 20 min before reperfusion and a maintenance dose of 20 μg/kg/min for the first 60 min of reperfusion. At the same time, the rats were post-treated for myocardial ischemia (30 s ischemia/30 s reperfusion administered 3 times within 3 min of the start of reperfusion). Reperfusion was allowed to occur for a total of 2.5 h.
6. DM Sham group: Diabetic rats were used for this group. Sutures were threaded without ligation.
7. DM I/R group: Diabetic rats were used for this group. I/R injury was induced as described in Myocardial ischaemia‒reperfusion model.
8. DM I-Post group: Diabetic rats were treated for myocardial ischemia (30 s ischemia/30 s reperfusion given 3 times within 3 min of the start of reperfusion). Each rat was reperfused for 2.5 h.
9. DM Nic group: Diabetic rats were administered a loading dose of 200 μg/kg nicorandil 20 min before reperfusion and a maintenance dose (20 μg/kg/min) was administered during the first 60 min of reperfusion. Then, the rats were reperfused for another 1.5 h (2.5 h total reperfusion time).
10. DM I-Post + Nic group: Diabetic rats were administered a loading dose of 200 μg/kg of nicorandil 20 min before reperfusion and a maintenance dose of 20 μg/kg/min for the first 60 min of reperfusion. At the same time, the rats were post-treated for myocardial ischemia (30 s ischemia/30 s reperfusion administered 3 times within 3 min of the start of reperfusion). Reperfusion was allowed to occur for a total of 2.5 h.

### Serum assay

After reperfusion, blood was collected from the rat abdominal aorta. The serum was obtained by centrifugation (4 °C, 1000 g, 10 min) and frozen at − 20 °C until further analysis. Serum creatine kinase-MB (CK-MB) levels were measured using the rat creatine kinase isozyme MB (CK-MB) ELISA kit (Elabscience, Wuhan, China). The detection range of the kit is 31.25-2000 pg/ml, the intra- and inter-batch coefficient of variation is less than 10%, and the minimum detection concentration is less than 18.75 pg/ml. Serum nitric oxide (NO) levels were measured using a rat nitric oxide ELISA kit (Elabscience, Wuhan, China). The detection range of the kit is 0.16-100 μmol/L, with intra- and inter-batch coefficients of variation less than 2.4% and 3.7%, respectively, and the minimum detection concentration less than 0.16 μmol/L.

### Reactive oxygen species (ROS) assay

At the end of reperfusion, rats were sacrificed. The heart was lavaged with 100 mL 0.9% saline and stored at 4 °C. Then, 50 mg of heart tissue was isolated from the below the ligation site and was assayed using a Rat Reactive Oxygen Species ELISA kit (Joln, Nanjing, China). The detection range of the kit is 1.0U/ml-80U/ml, with intra- and inter-batch coefficients of variation less than 9% and 11%, respectively, and the minimum detection concentration less than 1.0U/ml. Protein concentration (µg/µL) were determined using a BCA assay kit (Thermo, USA) to derive the mean level of ROS release (U/mg).

### Triphenyl tetrazolium chloride and Evens blue staining to determine the area of myocardial infarction

After reperfusion, the rat LAD vessels were again ligated and 1 ml of 1% Evans Blue staining solution was injected rapidly from the left ventricle to show the ischemic risk area (AAR). After clipping the heart (Heart samples shown in Additional file 1: Figure S2), the hearts were lavaged with 100 mL 0.9% saline at 4 °C and placed on ice at − 80 °C for 10–15 min. After freezing, the hearts were cut into 2-mm thick transverse sections. The transverse sections were placed in 1% 2,3,5-triphenyltetrazolium chloride (TTC; Sigma, USA) in 0.1 mM phosphate (1X PBS) buffer (pH 7.4) at 37 °C for 15–30 min. Then, the tissues were washed with 1X PBS buffer (3 times for 10 min each), and a bluish-purple color was seen in non-infarcted myocardium and no coloration in infarcted myocardium. It was then placed in 4% paraformaldehyde at 4 °C overnight. Images were analyzed using ImageJ data acquisition software (National Institutes of Health, Bethesda, MD, USA). Area measurement method: expressed as infarct area (IS) as a percentage of AAR (IS/AAR) and AAR as a percentage of total area (AAR/LV).

### TUNEL assay

After reperfusion, the hearts were then successively lavaged with 100 mL 0.9% saline and 50 mL 4% paraformaldehyde at 4 °C. After lavage, a cross Sect. 2 mm thick was cut perpendicular to the sagittal plane of the heart 2 mm below the ligature site. Fixed in 4% paraformaldehyde 3 days. Paraffin sections were prepared by dehydration and paraffin embedding. The prepared paraffin sections were analyzed using a TUNEL assay kit (Roche). In each slide, color images of five different fields were randomly captured and digitized. Cells that stained blue were normal cardiomyocytes and cells that stained brown were defined as TUNEL-positive cells. The apoptosis index (AI) was calculated as the number of TUNEL-positive cells/total number of cardiomyocytes × 100.

### Hematoxylin–eosin (HE) staining

After taking the paraffin sections prepared in the previous step of the experiment and staining them with HE staining kit (Roche), pathological sections were evaluated by a double-blinded pathologist. Lesions consisting of interstitial oedema, myofiber degeneration (i.e., myofiber swelling and myofibrillar lysis), and the formation of myocardial hypercontraction bands were graded according to their severity (0 = no lesion, 1 = mild, 2 = moderate, 3 = marked) and distribution (0 = no lesion, 1 = focal lesion, 2 = multifocal lesion, 3 = diffuse lesion). The mean score for each variable was calculated for each heart, and the group mean score was calculated [19].

### Wheat germ agglutinin (WGA) immunofluorescence staining

Plaster sections were made by taking the previous step. The sections were blotted dry with absorbent paper, the heart sections were circled with a histochemical pen, and WGA staining solution (iFluor 488 wheat germ agglutinin conjugate, ATT-Bioquest, USA) diluted 200 times in PBS was added dropwise to the circles, and then incubated for 30 min at 37 °C in a constant temperature chamber protected from light. After incubation, the slides were washed 3 times with PBS (5 min/time) and fluorescence quenching agent was added dropwise for 5 min. After washing, the slides were washed 3 times with PBS (5 min/time), anti-fluorescence quenching sealer was added dropwise in the circle, and the slides were covered with coverslips and sealed with nail polish and stored in a wet box at 4 °C. The digital section scanning system of the Department of Pathology, The Affiliated Hospital of Qingdao University was used to take pictures for observation and analyze the cross-sectional area of cells and the degree of tissue lesions.

### Western blot

The rats were sacrificed 15 min after the resuscitation. Then, the heart was lavaged with 100 mL 0.9% saline at 4 °C. Heart tissue from the anterior wall of the left ventricle was isolated from the AAR and used for western blot. The extracted heart tissue was homogenized with RIPA lysis buffer (Elabscience, Wuhan, China). The tissue was then centrifuged at 15,000 × *g* for 10 min at 4 °C. The supernatant was collected, and the protein concentration was determined using a BCA assay kit (Thermo, USA). The samples were separated using 10% SDS-PAGE gels (10 μg/well). The protein bands were transferred onto nitrocellulose membranes (Merck Millipore, USA). The membranes were then blocked in 5% skim milk for 2 h and incubated overnight at 4 °C with the following primary antibodies: p-PI3K (#4228), PI3K (#4257); p-GSK3β (#5558), GSK3β (#12,456); p-Akt (#4060), Akt (#4691); p-mToR (#5536), mToR (#2983); p-eNOS (#9574), eNOS (#32,027); GAPDH (#5174) (all rabbit, 1:1000, Cell Signaling Technology, USA). horseradish peroxidase (HRP)-labeled goat anti-rabbit IgG (1; 10,000, Absin, Shanghai, China) was used as the secondary antibody. Target bands were detected using chemiluminescent ECL (Merck Millipore, USA) and visualized using an Amersham Imager 600 (GE Healthcare, Little Chalfont, UK). The images were analyzed using ImageJ data acquisition software.

### Statistical analysis

The monitoring data were statistically analyzed using GraphPad Prism9 (La Jolla, CA, USA) and Image J. All data were expressed as mean ± standard error of mean (SEM). T-test was used for statistical analysis of differences between two groups; Differences between multiple groups were analyzed using one-way ANOVA, and Bonferroni multiple comparison test was used for comparison between groups. Statistical significance was set at *P* < 0.05.

## Results

### Confirming the phenotype of type 2 diabetic rats

**Fig. 1:**
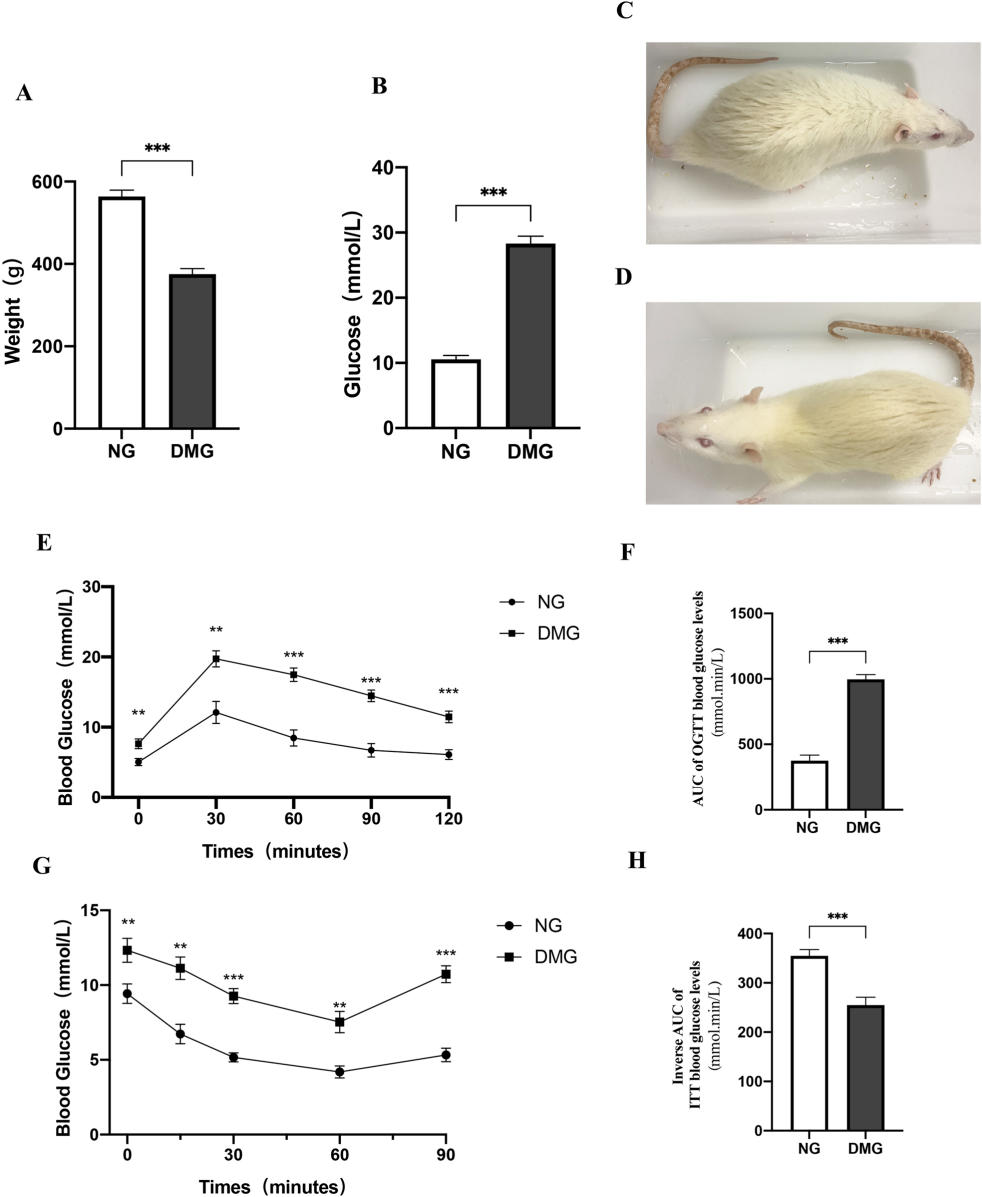
Body weight and blood glucose of rats in each experimental group. **A** Plasma glucose (n = 20), **B** Body weight (n = 20). **C** A representative nondiabetic rat. **D** A representative diabetic rat. **E** Glucose levels during OGTT. **F** Glucose area under the curve (AUC) during OGTT. **G** Glucose levels during ITT. **H** Glucose area under the curve (AUC) during ITT. **P < 0.01, ***P < 0.001. NG: normal group. DMG: diabetes mellitus group. Data are expressed as mean ± standard error of the mean (SEM) and were analysed by T test

As shown in Fig. 1, we induced type 2 diabetes using a single intraperitoneal injection of STZ in combination with a high-fat, high-sugar diet. After 12 weeks, the body weight of DMG rats decreased by 35% compared with that of NG rats without STZ injection (***P < 0.001, Fig. 1A). Blood glucose in the DMG rats increased 2.9-fold (***P < 0.001, Fig. 1B). DMG rats showed significant wasting, yellowing and darkening of the fur and possible blindness due to diabetic retinopathy (Fig. 1C, D).

While patients and animals with T2DM do not necessarily suffer from obesity, being overweight is a common phenotype for them. To further verify the presence of insulin resistance in DMG rats, we used OGTT and ITT to verify whether DMG rats are consistent with the T2DM phenotype [20]. The OGTT (Fig. 1E) showed that after administration of a glucose loading dose to NG rats, glucose peaked at 30 min and decreased to basal levels after 120 min, indicating normal glucose elimination. In marked contrast, glucose elimination in DMG rats was significantly delayed after reaching a peak at 30 min and was significantly higher than NG at 60 and 90 min (***P < 0.001). It remained above basal metabolic levels at 120 min. In addition, fasting glucose levels were higher in the DMG group of rats fed a high-fat diet as a result of the OGTT process. We further analysed whether there was a difference by correcting the baseline and calculating the area under the curve (AUC).We found a higher AUC in the DMG group rats, further confirming the presence of impaired glucose tolerance in the DMG (***P < 0.001, Fig. 1F). Subsequently, to examine the insulin sensitivity of DMG rats, we performed an ITT experiment (Fig. 1G). In this experiment, the degree of decrease in blood glucose concentration after insulin injection represented the efficiency of systemic insulin action. Blood glucose in NG and DMG rats decreased rapidly within 15 and 30 min, reaching a minimum after 60 min. However, at all time points during ITT, the overall blood glucose of DMG rats remained higher than that of NG rats (***P < 0.001), indicating a significantly impaired decrease in blood glucose levels and the presence of significant insulin resistance in DMG rats. In addition, as there was a difference in blood glucose between the two groups at minute 0 during the ITT, we further analysed whether there was a difference by calculating the AUC at the corrected inverse baseline. Basal blood glucose levels (time point 0) were subtracted from the blood glucose values obtained at each subsequent time point, and these values were multiplied by -1 before calculating each AUC. We found that the AUC was significantly higher in the DMG than in the NG (***P < 0.001, Fig. 1H), indicating reduced sensitivity to insulin in the DMG rats. Therefore, we conclude that both OGTT and ITT respond to the presence of significant insulin resistance and impaired glucose tolerance in DMG rats.

### Diabetes attenuates the cardioprotective effect of I-Post and nicorandil on myocardial I/R injury

**Fig. 2:**
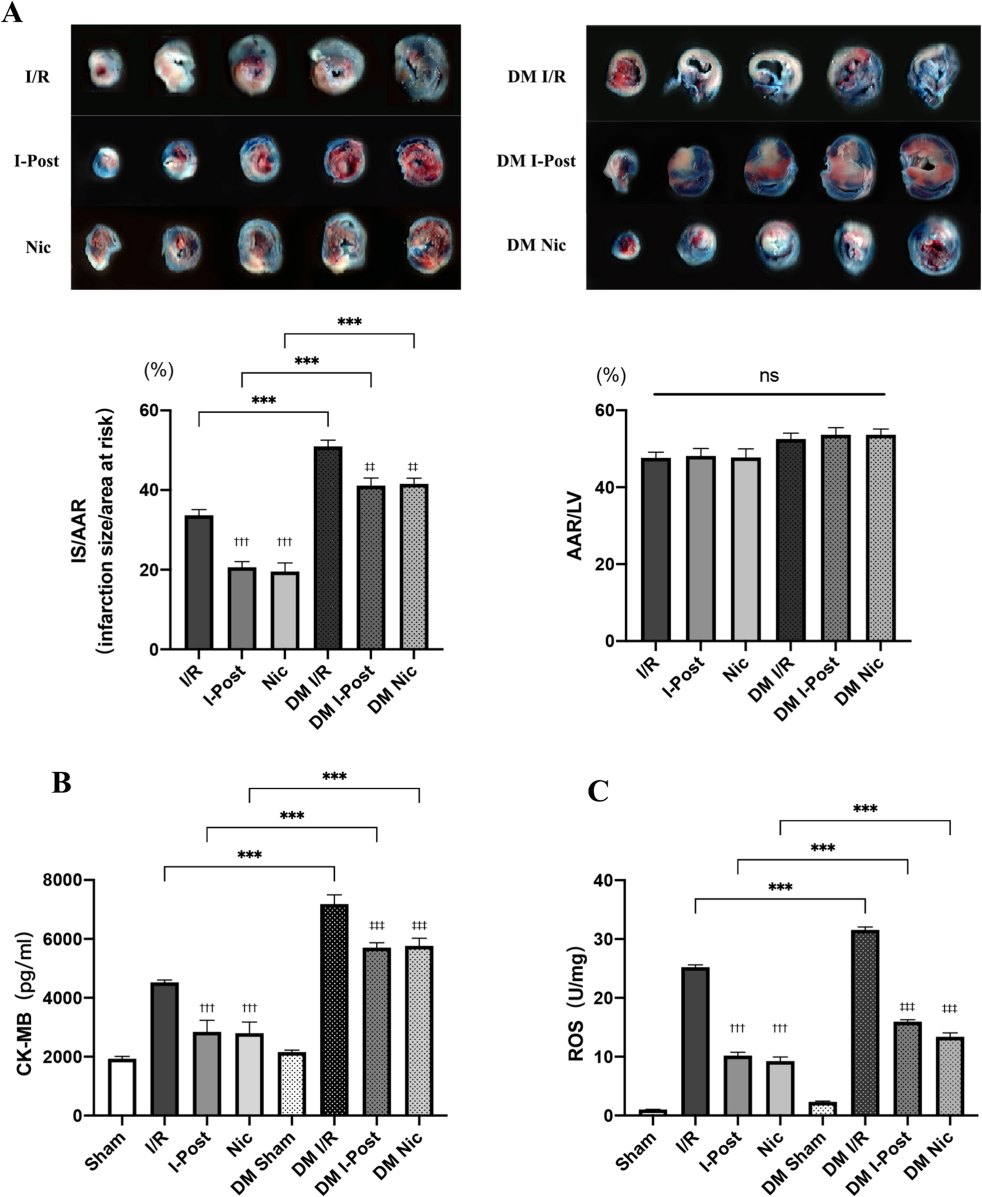
Diabetes impairs the cardioprotective effects of I-Post and nicorandil, and the cardioprotective effect of the combination treatment on myocardial I/R injury in diabetic rats. **A** Representative sections of TTC/Evans Blue stained heart tissue subjected to 30 min myocardial ischemia followed by 2.5 h of reperfusion and infarct area histogram analysis (n = 5). **B** CK-MB plasma levels in rats treated with I-Post or nicorandil (n = 6). **D** ROS levels in myocardial tissue in rats treated with I-Post or nicorandil (n = 6). Compared with I/R: ^†††^*P* < 0.001; Compared with DM I/R: ^‡‡‡^*P* < 0.001; Comparison between nondiabetic and diabetic groups applying the same treatment: ***P < 0.001. Data represent the mean ± SEM. Data were analysed using one-way ANOVA and Bonferroni multiple comparisons test. Sham, sham surgery; I/R, ischaemia‒reperfusion; I-Post, ischaemia postconditioning; Nic, nicorandil; DM, diabetes mellitus

**Fig. 3:**
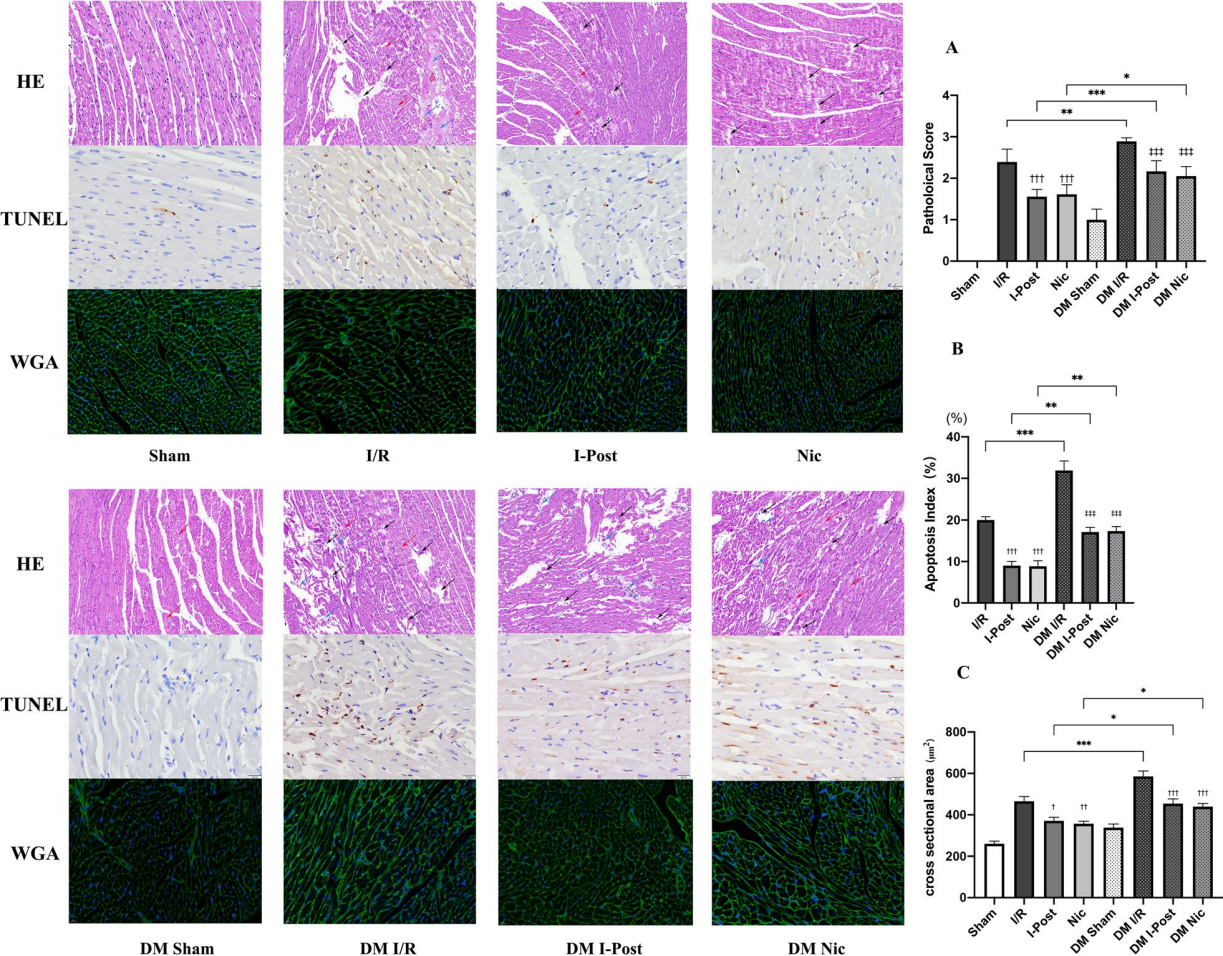
Alterations in myocardial tissue structure in diabetic rats after I-Post or nicorandil. **A** Representative cross-sectional HE-stained pathological section of the heart, and comparison of pathological damage scores (n = 6). Red arrows indicate cellular oedema as well as myocardial fibre swelling; black arrows represent myofiber lysis; blue arrows represent inflammatory cell infiltration. **B** Representative TUNEL-stained sections of the heart, and apoptotic index of cardiomyocytes in each group (n = 5). Brown nuclei indicate TUNEL-positive nuclei (marked by red arrow). **C** Representative cross-sectional WGA staining of the heart, and quantitative data on the cross-sectional area of cardiomyocytes (n = 6). Compared with I/R: ^†††^*P* < 0.001; Compared with DM I/R: ^‡‡‡^*P* < 0.001; Comparison between nondiabetic and diabetic groups applying the same treatment: ***P < 0.001, **P < 0.01, *P < 0.05,. Data represent the mean ± SEM. Data were analysed using one-way ANOVA and Bonferroni multiple comparisons test. Sham, sham surgery; I/R, ischaemia‒reperfusion; I-Post, ischaemia postconditioning; Nic, nicorandil; DM, diabetes mellitus

As shown in Fig. 2, we first assessed the extent of myocardial injury in diabetic I/R rats. TTC and Evans blue staining enabled visualization of the area of myocardial injury, and we found that the infarct area was 20% larger in diabetic rats (50.96% ± 1.59%) than in nondiabetic rats (33.65% ± 1.44%) ( ***P < 0.001, Fig. 2A). CK-MB and ROS are important indicators of the degree of myocardial injury, and we observed that plasma CK-MB levels and myocardial tissue ROS levels in diabetic rats after I/R compared with nondiabetic rats were significantly higher (***P < 0.001, Fig. 2B, C), which may reflect the higher sensitivity of diabetic patients to myocardial I/R injury. Furthermore, as shown in Fig. 3, we also observed by HE staining that diabetic rats had more severe histomorphological lesions than nondiabetic rats, including extensive myocardial cell ooedema, massive inflammatory cell infiltration into the myocardial interstitial space, disorganized myocardial fibre structure, and diffuse foci of necrosis (Fig. 3A). By TUNEL staining, we found that the number of TUNEL-positive nuclei was significantly increased, and the apoptotic index of cardiomyocytes was significantly increased (***P < 0.001, Fig. 3B). In addition, we found by WGA staining that the cross-sectional area of cardiomyocytes was significantly increased in diabetic I/R rats (585.68 ± 26.119 μm^2^) compared with nondiabetic I/R rats (465.83 ± 22.251 μm^2^) (***P < 0.001, Fig. 3C), and cardiomyocyte morphology was significantly altered. All of these results indicated an increase in myocardial necrosis in diabetic rats after I/R.

The application of therapy with I-Post (20.62% ± 1.42%) or nicorandil (19.52% ± 2.17) resulted in a significant reduction in IS/AAR in nondiabetic rats compared with I/R (33.65% ± 1.44%) (all ^†††^*P* < 0.001, Fig. 2A) but failed to achieve the same therapeutic effect in diabetic I/R rats. As shown in Fig. 2, although myocardial IS/AAR was reduced by 9% in diabetic I/R rats (50.96% ± 1.59%) after I-Post (41.13% ± 2.17%) or nicorandil treatment (41.54 ± 1.45%) (^‡‡^*P* < 0.01, Fig. 2A), the degree of myocardial injury was still higher than that in nondiabetic rats after the same treatment, which may indicate that diabetes impairs the cardioprotective effects of I-Post as well as nicorandil. Similar to the TTC/Evans blue staining results, although treatment with I-Post or nicorandil reduced plasma CK-MB levels and myocardial tissue ROS levels in diabetic I/R rats (^‡‡‡^*P* < 0.001, Fig. 2B, C), these measures of myocardial injury remained higher than those in nondiabetic rats (***P < 0.001, Fig. 2B, C).

In addition, we systematically assessed the altered myocardial histomorphology using pathology scoring criteria (Fig. 3A) and calculated the apoptotic index of cardiomyocytes (Fig. 3B), as well as investigated the morphological changes of diabetic cardiomyocytes after I-Post and nicorandil treatment by WGA staining (Fig. 3C). We found that although I-Post or nicorandil treatment attenuated cardiac I/R injury in diabetic rats to some extent, interstitial inflammatory cell infiltration as well as myocardial fibre breaks were still visible in pathological sections as focal necrotic lesions compared with nondiabetic I/R rats after the same treatment (*P < 0.05, Fig. 3A). Scattered TUNEL-positive nuclei were still visible in TUNEL-stained sections (**P < 0.01, Fig. 3B). In addition, we also found abnormal cardiomyocyte morphology with significant cell swelling after I-Post or nicorandil treatment in diabetic I/R rats compared with nondiabetic I/R rats by WGA staining (*P < 0.05, Fig. 3C). This result shows that the benefit of I-Post or nicorandil alone in treating diabetic myocardial I/R injury is limited. Therefore, we conclude that diabetes mellitus diminishes the therapeutic effect of I-Post and nicorandil.

### Diabetes affects the cardioprotective effect of I-Post or nicorandil on myocardial I/R injury in diabetic rats by weakening the PI3K/Akt signalling pathway

**Fig. 4:**
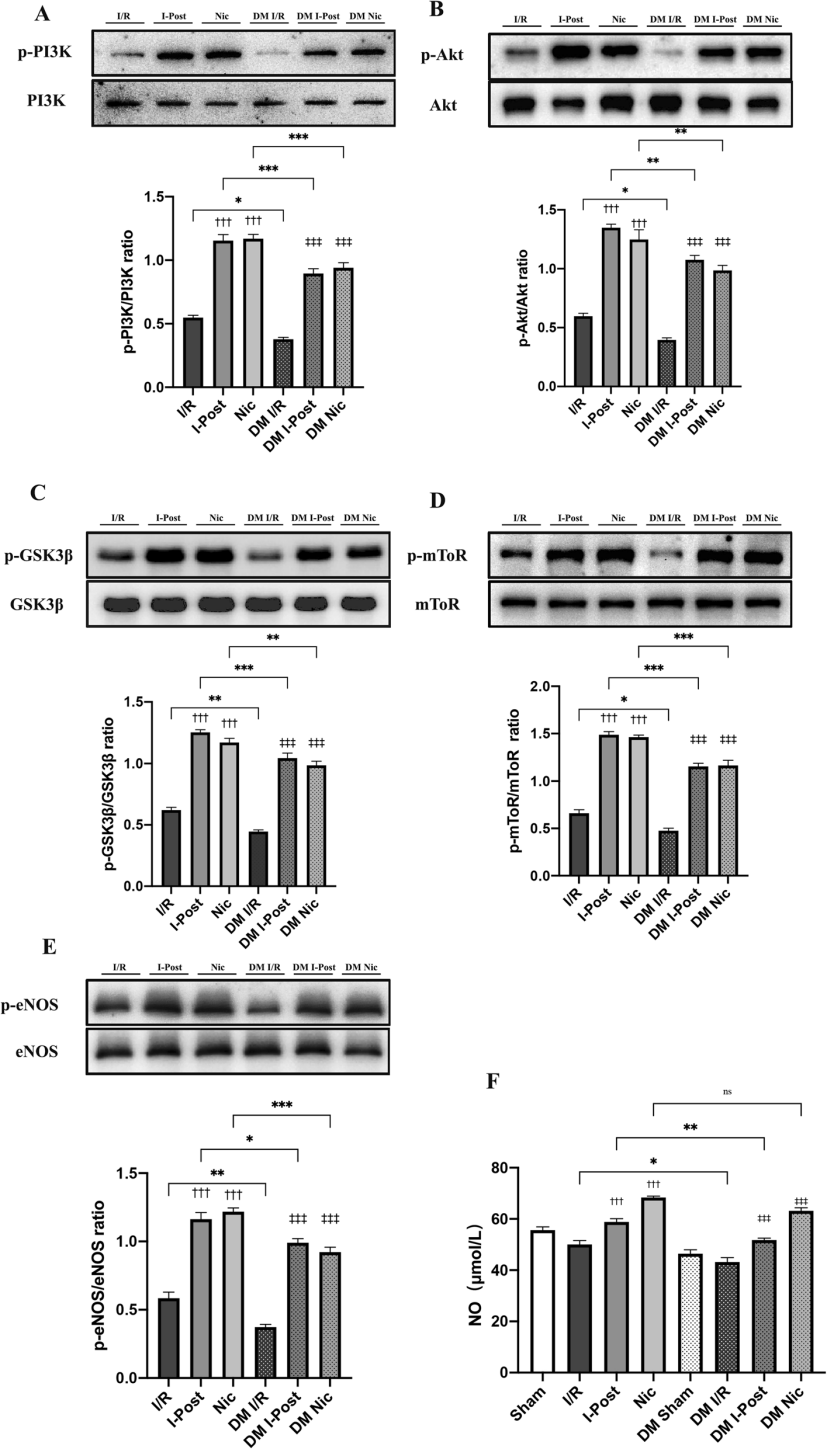
Alteration of PI3K/Akt signalling pathway in diabetic rats after I-Post or nicorandil treatment alone **A** Representative western blot showing total expression and the phosphorylation state. Comparison of p-PI3K expression levels in myocardial tissue of rats treated with I-Post or nicorandil (n ≥ 3). **B** Comparison of p-Akt expression levels in myocardial tissue of rats treated with I-Post or nicorandil (n ≥ 3). **C** Comparison of p-GSK3βexpression levels in myocardial tissue of rats treated with I-Post or nicorandil (n ≥ 3). **D** Comparison of p-mTOR expression levels in myocardial tissue of rats treated with I-Post or nicorandil (n ≥ 3). **E** Comparison of p-eNOS expression levels in myocardial tissue of rats treated with I-Post or nicorandil (n ≥ 3). **F** NO plasma levels in rats treated with I-Post or nicorandil (n = 6). Compared with I/R: ^†††^*P* < 0.001; Compared with DM I/R: ^‡‡‡^*P* < 0.001; Comparison between nondiabetic and diabetic groups applying the same treatment: ***P < 0.001, **P < 0.01, *P < 0.05, ^ns^*P* > 0.05. Data represent the mean ± SEM. Data were analysed using one-way ANOVA and Bonferroni multiple comparisons test. Sham, sham surgery; I/R, ischaemia‒reperfusion; I-Post, ischaemia postconditioning, Nic, nicorandil; DM, diabetes mellitus

As mentioned previously, diabetes impaired the protective effect of I-Post as well as nicorandil on diabetic myocardial I/R injury. To investigate the related mechanism, we performed Western blotting to detect changes in the relevant cardioprotective signalling pathways. The previous studies have shown that I-Post and nicorandil can exert cardioprotective effects through activation of the PI3K/Akt signalling pathway [7, 14]. Our study also confirmed that in nondiabetic I/R rats, after I-Post and nicorandil treatment, the expression of phosphorylated PI3K (p-PI3K), phosphorylated Akt (p-Akt), phosphorylated mTOR (p-mTOR), phosphorylated GSK3β (p-GSK3β) and phosphorylated eNOS (p-eNOS) was significantly increased in rat hearts (all^†††^*P* < 0.001, Fig. 4). However, in diabetic rats, although the expression of p-PI3K, p-Akt, p-GSK3, p-mToR, and p-eNOS was significantly increased in diabetic I/R rats after I-Post or nicorandil treatment (all ^‡‡‡^*P* < 0.001, Fig. 4), these key proteins of the PI3K/Akt signalling pathway failed to reach the same level of phosphorylation in diabetic I/R rats as in nondiabetic rats with the same level of phosphorylated expression (*P < 0.05, Fig. 4). This may be the main reason why diabetes impairs the cardioprotective effects of I-Post and nicorandil in I/R rats.

In addition, the level of nitric oxide (NO), an important endogenous vasodilator, may reflect the cardioprotective effect of I-Post and nicorandil. We found that in nondiabetic I/R rats, the plasma levels of NO were significantly higher after treatment with I-Post or nicorandil (^†††^*P* < 0.001, Fig. 4F). However, in diabetic rats, although the plasma levels of NO were significantly increased in diabetic I/R rats after I-Post treatment (^‡‡‡^*P* < 0.001, Fig. 4F), the levels were still lower than those in nondiabetic I/R rats after I-Post treatment (**P < 0.01, Fig. 4F). This result also reflects that diabetes impaired the protective effect of I-Post on myocardial I/R injury.

Furthermore, we unexpectedly found that plasma NO levels were not significantly different in diabetic I/R rats after treatment with nicorandil compared with nondiabetic I/R rats, which may indicate that diabetes does not affect the NO-promoting effect of nicorandil (^ns^*P* > 0.05, Fig. 4F).

### Protective effect of I-Post combined with nicorandil in diabetic rats with myocardial infarction

As described previously, diabetes impaired the cardioprotective effect of I-Post or nicorandil on myocardial I/R injury, suggesting that one therapeutic measure alone does not provide a good therapeutic effect and that combination therapy may be a potential treatment modality for such diseases. Therefore, we applied the therapeutic measure of I-Post combined with nicorandil to explore its rationality and feasibility.

**Fig. 5:**
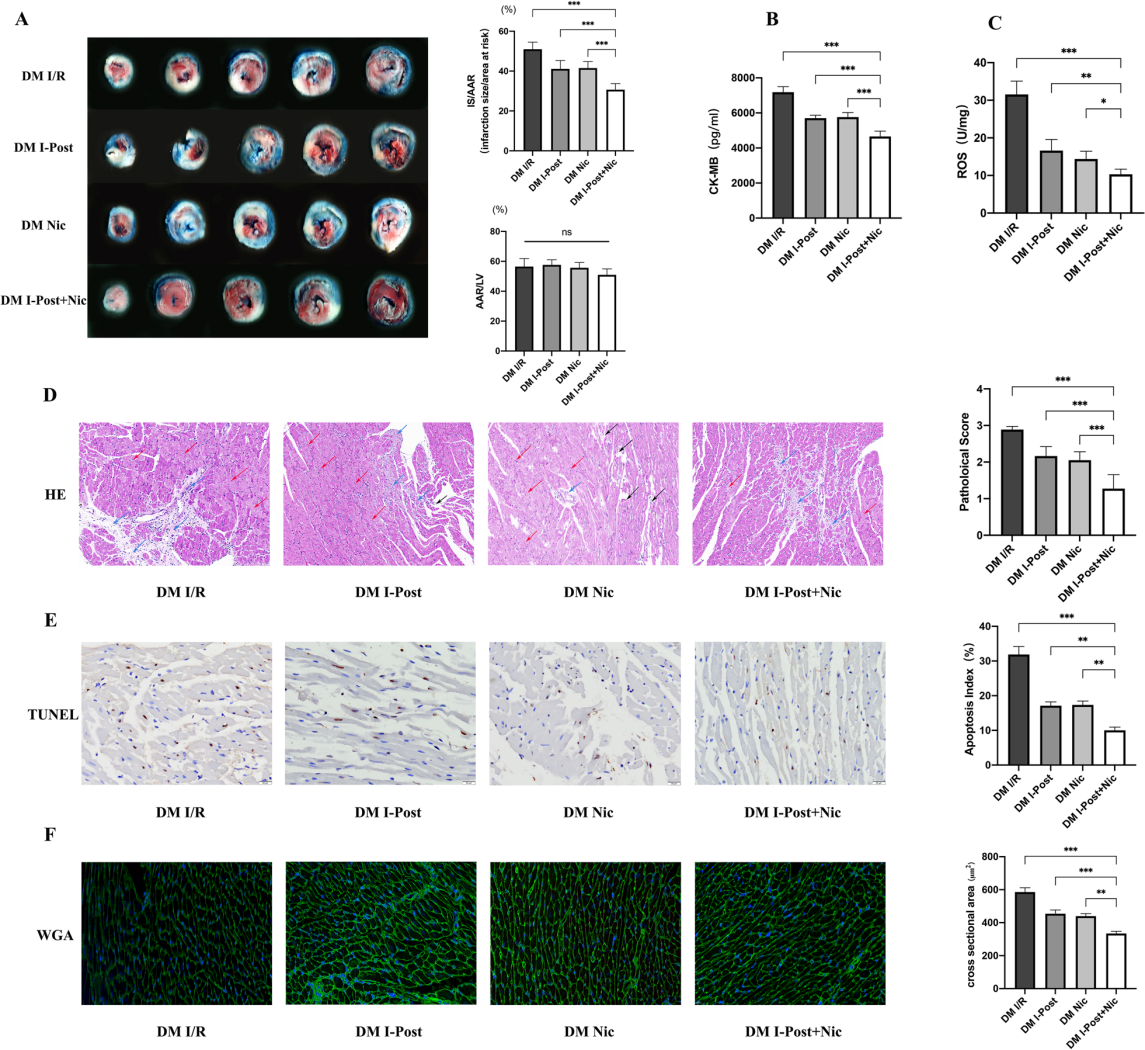
Protective effect of I-Post combined with nicorandil on myocardial I/R injury in diabetic rats **A** Representative sections of TTC/Evans Blue stained heart tissue subjected to 30 min myocardial ischemia followed by 2.5 h of reperfusion and infarct area histogram analysis (n = 5). **B** CK-MB plasma levels in rats treated with I-Post combined with nicorandil (n = 6). **C** ROS levels in myocardial tissue in rats treated with I-Post combined with nicorandil (n = 6). **D** Representative cross-sectional HE-stained pathological section of the heart, and comparison of pathological damage scores (n = 6). Red arrows indicate cellular oedema as well as myocardial fiber swelling; black arrows represent myofiber lysis; blue arrows represent inflammatory cell infiltration. **E** Representative TUNEL-stained sections of the heart, and apoptotic index of cardiomyocytes in each group (n = 5). **F** Representative cross-sectional WGA staining of the heart, and quantitative data on the cross-sectional area of cardiomyocytes (n = 6). Compared with I-Post + Nic: ***P < 0.001, **P < 0.01, *P < 0.05. Data represent the mean ± SEM. Data were analysed using one-way ANOVA and Bonferroni multiple comparisons test. I/R, ischaemia‒reperfusion; I-Post, ischaemia postconditioning; Nic, nicorandil; I-Post + Nic: Combination of I-Post and Nicorandil treatment measures; DM, diabetes mellitus

As shown in Fig. 5, we observed that the IS/AAR was reduced by 20% in diabetic rats treated with I-Post combined with nicorandil (30.67% ± 1.38% vs. 50.96% ± 1.58%, ***P < 0.001, Fig. 5A), which was more effective in reducing IS/AAR than in diabetic rats treated with I-Post (41.13% ± 2.17%) or nicorandil (41.54 ± 1.45%) alone (all ***P < 0.001, Fig. 5A). Moreover, the plasma levels of CK-MB and myocardial tissue levels of ROS in diabetic I/R rats after I-Post combined with nicorandil treatment were significantly reduced (all **^*^P < 0.001, Fig. 5B, C), which was a significant improvement compared with I-Post or nicorandil alone (all ^***^P < 0.001, Fig. 5B, C).

By HE staining, we observed a more regular alignment of myocardial fibres after the combination treatment, and no localized necrotic lesions were observed (^***^P < 0.001, Fig. 5D). The pathology score of I-Post combined with nicorandil was lower than that of I-Post (***P < 0.001, Fig. 5D) or nicorandil alone (^***^P < 0.001, Fig. 5D). WGA staining and TUNEL staining yielded similar results. I-Post combined with nicorandil resulted in a significant reduction in apoptotic cells (^***^P < 0.001, Fig. 5E), a decrease in cardiomyocyte ooedema, and a significant reduction in cardiomyocyte cross-sectional area (344.45 ± 13.434 μm^2^ vs. 585.68 ± 26.119 μm^2^, ^**^*P < 0.001, Fig. 5F). Compared with diabetic I/R rats treated with I-Post (454.64 ± 13.434 μm^2^) or nicorandil (439.95 ± 15.087 μm^2^) alone (all ***P < 0.001, Fig. 5F), the benefits of the combination treatment were greater.

These results suggest that in diabetic rats, treatment with I-Post combined with nicorandil yielded better cardioprotection against myocardial I/R injury.

### I-Post combined with nicorandil in diabetic I/R rats can have a superimposed effect on activation of the PI3K/Akt signalling pathway

**Fig. 6:**
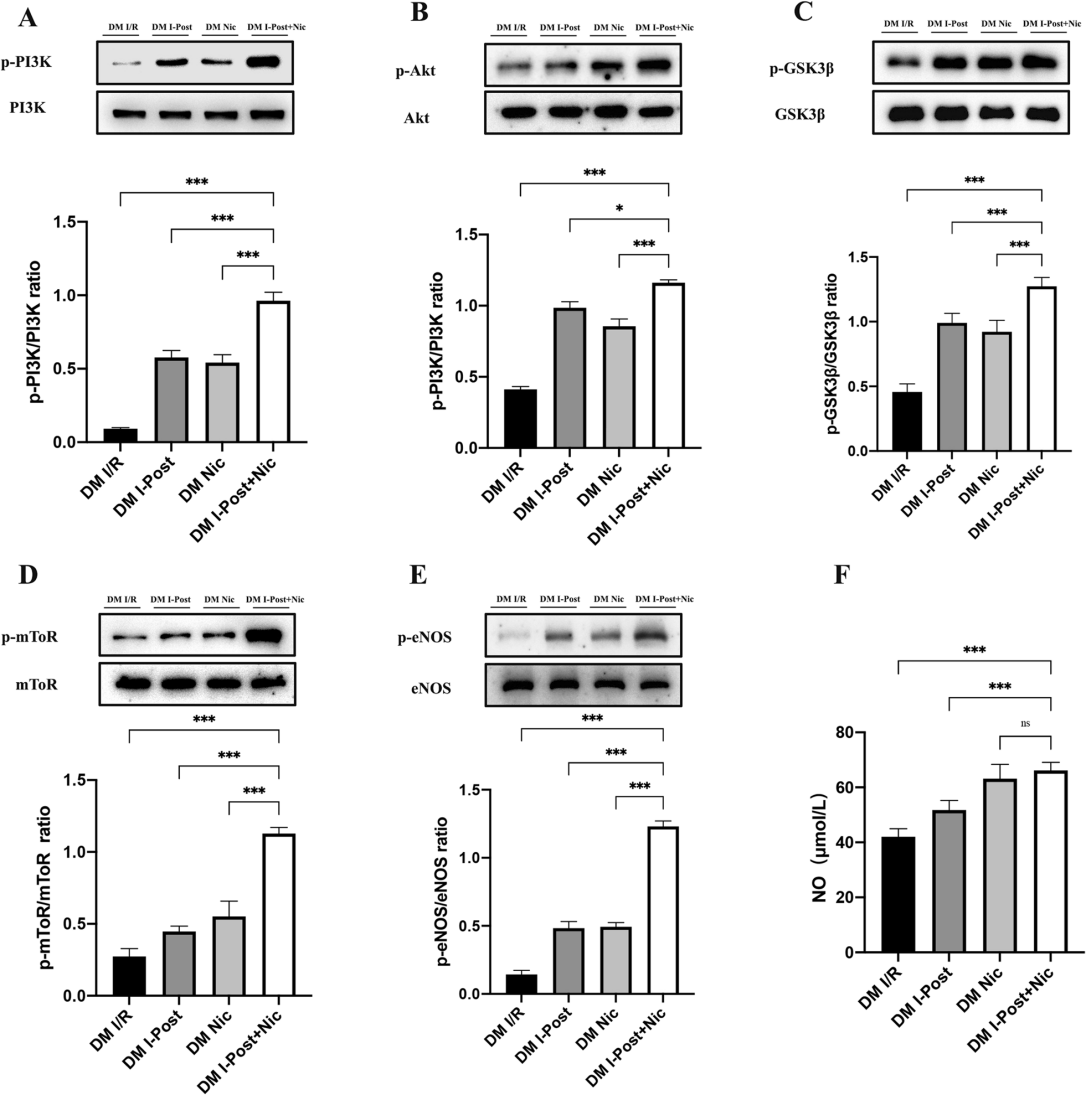
Alteration of PI3K/Akt signalling pathway in diabetic rats after I-Post combined with nicorandil treatment alone **A** Representative western blot showing total expression and the phosphorylation state. Comparison of p-PI3K expression levels in myocardial tissue of rats treated with I-Post combined with nicorandil (n ≥ 3). **B** Comparison of p-Akt expression levels in myocardial tissue of rats treated with I-Post combined with nicorandil (n ≥ 3). **C** Comparison of p-GSK3βexpression levels in myocardial tissue of rats treated with I-Post combined with nicorandil (n ≥ 3). **D** Comparison of p-mTOR expression levels in myocardial tissue of rats treated with I-Post combined with nicorandil (n ≥ 3). **E** Comparison of p-eNOS expression levels in myocardial tissue of rats treated with I-Post combined with nicorandil (n ≥ 3). **F** NO plasma levels in rats treated with I-Post combined with nicorandil (n = 6). Compared with I/R: ^†††^*P* < 0.001; Compared with DM I/R: ^‡‡‡^*P* < 0.001; Compared with I-Post + Nic: ***P < 0.001, **P < 0.01, *P < 0.05, ^ns^*P* > 0.05. Data represent the mean ± SEM. Data were analysed using one-way ANOVA and Bonferroni multiple comparisons test. Sham, sham surgery; I/R, ischaemia‒reperfusion; I-Post, ischaemia postconditioning; Nic, nicorandil; I-Post + Nic: Combination of I-Post and Nicorandil treatment measures; DM, diabetes mellitus

Diabetic I/R rats treated with I-Post in combination with nicorandil had significantly increased expression levels of p-PI3K, p-Akt and its downstream effectors p-GSK3b, p-mToR and p-eNOS in myocardial tissue (all ***P < 0.001, Fig. 6). The phosphorylation levels of all these proteins were higher than those of diabetic I/R rats treated with I-Post (all *P < 0.05, Fig. 6) or nicorandil (all ^***^P < 0.001, Fig. 6) alone.

In addition, the combination treatment significantly increased plasma NO levels in diabetic I/R rats (***P < 0.001, Fig. 6F) and was significantly beneficial for increasing plasma NO levels compared with diabetic I/R rats treated with I-Post alone. However, we also found no significant difference in plasma NO levels between the combination treatment group and diabetic I/R rats treated with nicorandil alone (^ns^*P* > 0.05, Fig. 6F).

### Activation of the PI3K/Akt signalling pathway in diabetic I/R rats by I-Post combined with nicorandil is inhibited by the PI3K phosphorylation inhibitor wortmannin

**Fig. 7:**
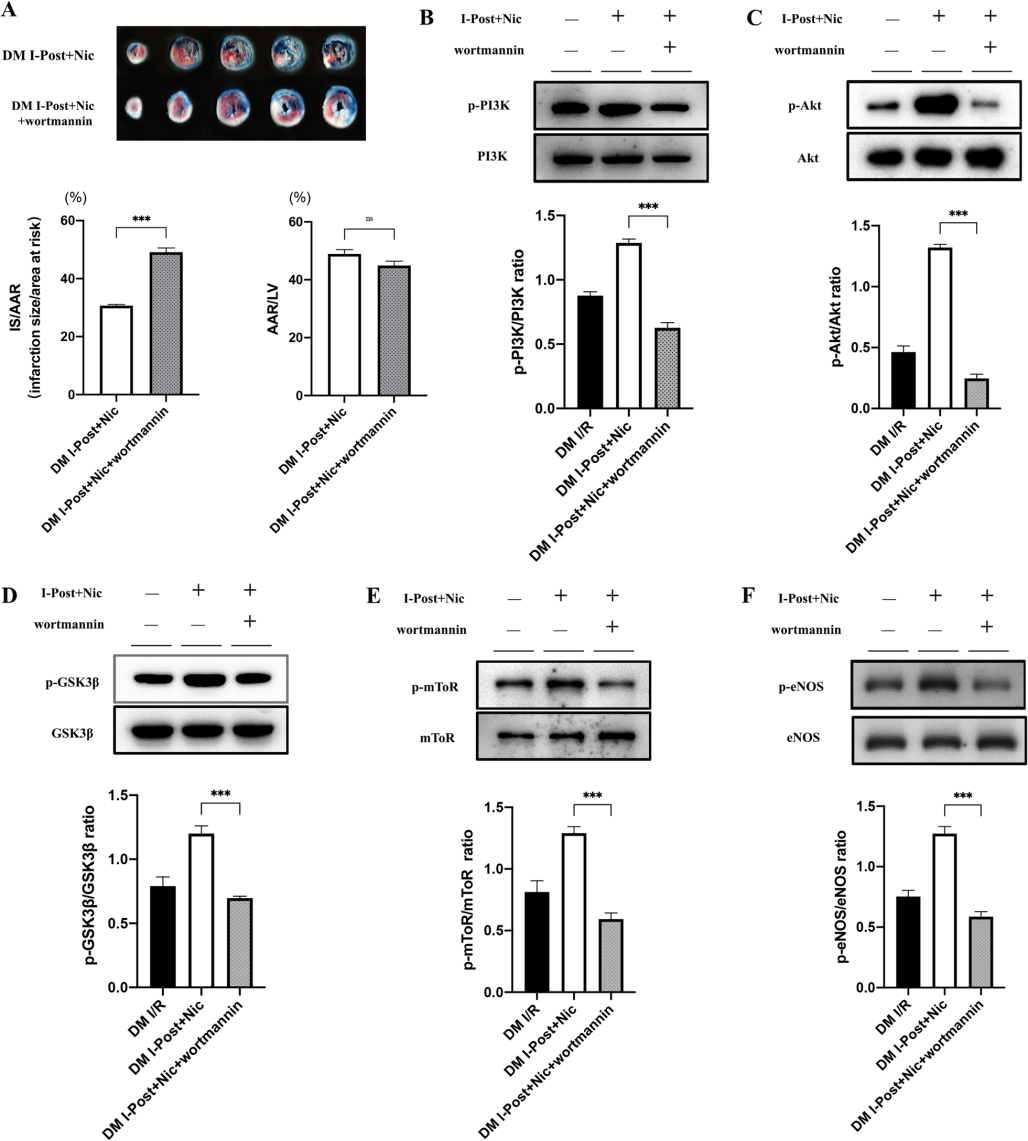
Activation of PI3K/Akt signalling pathway by I-Post combined with nicorandil is hindered by PI3K phosphorylation inhibitor wortmannin **A** Representative sections of TTC/Evans Blue stained heart tissue subjected to 30 min myocardial ischemia followed by 2.5 h of reperfusion and infarct area histogram analysis (n = 5). **B** Representative western blot showing total expression and the phosphorylation state, Comparison of p-PI3K expression levels in myocardial tissue of rats (n ≥ 3). **C** Comparison of p-Akt expression levels in myocardial tissue of rats (n ≥ 3). **D** Comparison of p-GSK3βexpression levels in myocardial tissue of rats (n ≥ 3). **E** Comparison of p-mTOR expression levels in myocardial tissue of rats(n ≥ 3). **F** Comparison of p-eNOS expression levels in myocardial tissue of rats (n ≥ 3). Compared with I-Post + Nic + wortmannin: ***P < 0.001. Data represent the mean ± SEM. Data were analysed using T test, one-way ANOVA and Bonferroni multiple comparisons test. I/R, ischaemia‒reperfusion; I-Post + Nic, ischaemia postconditioning, combined with nicorandil; wortmannin, PI3K phosphorylation inhibitor; DM, diabetes mellitus

We found that I-Post combined with nicorandil treatment may have a superimposed effect on the activation of the PI3K/Akt signalling pathway, which has a better cardioprotective effect on reducing myocardial I/R injury in diabetic I/R rats. To verify the mechanism of its cardioprotective effect, we applied the PI3K phosphorylation inhibitor wortmannin before treatment with I-Post combined with nicorandil in diabetic I/R rats, observed the extent of myocardial injury and analysed the expression of related protein phosphorylation in the PI3K/Akt signalling pathway. After TTC/Evans Blue staining, it was found that the application of wortmannin impeded the protective effect of I-Post combined with nicorandil on the myocardium of diabetic I/R rats (30.67% ± 1.38% vs. 49.08 ± 1.47%, ***P < 0.001, Fig. 7A) and that wortmannin significantly reduced the phosphorylation of PI3K and p-Akt (all ***P < 0.001, Fig. 7), which affected the activation of downstream target proteins such as eNOS, GSK3b and mToR (all ***P < 0.001, Fig. 7). We confirmed that the therapeutic measures of I-Post combined with nicorandil act through the PI3K/Akt signalling pathway.

## Discussion

Diabetes mellitus, a metabolic disease characterized by hyperglycaemia and endogenous insulin hypersecretion or resistance, is an independent risk factor for cardiovascular disease, and a large number of diabetic patients die suddenly due to AMI. Although timely reperfusion therapy is important to save diabetic AMI patients, diabetic patients exhibit a high susceptibility to myocardial I/R injury, which leads to loss of endogenous cardioprotective mechanisms and mitochondrial dysfunction [12]. In addition, the physiological balance of NO is altered in the hyperglycaemic state caused by diabetes [21] and leads to apoptosis triggered by massive production of ROS [22, 23]. In this study, we found that the regimen of I-Post combined with nicorandil was more cardioprotective in the treatment of diabetic myocardial I/R injury. The reasons are as follows: (1) diabetes impaired the efficacy of I-Post or nicorandil alone in the treatment of myocardial I/R injury. (2) Further mechanistic studies suggest that diabetes inhibited the activation of the PI3K/Akt signalling pathway by I-Post and nicorandil, resulting in depletion of endogenous cardioprotective mechanisms. (3) The treatment regimen of I-Post combined with nicorandil was more effective in activating the PI3K/Akt signalling pathway in diabetic I/R rats and had a superimposed effect. Combining the two therapeutic measures can provide better therapeutic effects in limiting diabetic myocardial I/R injury when a single therapeutic regimen is weakened.

### Diabetes impairs the cardioprotective effect of I-Post on myocardial I/R injury

In the present study, although the myocardial infarct area and the degree of myocardial injury were somewhat improved in diabetic I/R rats treated with I-Post, they still did not achieve therapeutic effects similar to those of nondiabetic I/R rats after the same treatment. Further mechanistic studies showed that diabetes impaired the activation of I-Post on the PI3K/Akt signalling pathway, thus affecting the protective effect of myocardial I/R injury in diabetic rats.

Recent large clinical trials, such as the POSTEMI (ST elevation postmyocardial infarction management) and DANAMI-3-iPOST (Third Danish study of optimal acute treatment of patients with ST-segment elevation myocardial infarction–ischaemic postconditioning) studies [24, 25], showed that I-Post does not reduce the myocardial infarction size, nor does it reduce mortality or heart failure hospitalization rates in patients with ST-segment elevation myocardial infarction undergoing emergency PCI. Therefore, this may explain the failure of I-Post in clinical studies. The actual clinical situation of patients is more complex, as their age and concomitant diseases, such as hypertension, diabetes and hyperlipidaemia, may affect the cardioprotective effect of I-Post. Although effective in animal studies, patients encountered in clinical work often have multiple comorbidities and, therefore, have limited impact in humans. In addition, repeated balloon dilation-reperfusion with I-Post during clinical interventions may cause thrombus dislodgement and lead to coronary microvascular embolization, thus affecting the efficacy of reperfusion therapy. Based on our results and those of large-scale clinical studies, we conclude that the complexity and severity of diabetes combined with AMI diminish the cardioprotective effect of I-Post on myocardial I/R injury and that a single treatment regimen may not achieve the desired therapeutic effect. In addition, the complexity of severe disease often compromises the therapeutic potential of available treatment options. As a result, current efforts to treat these diseases have gradually shifted from a focus on monotherapy to combination or multiple therapies [26].

Therefore, we concluded that the addition of drugs with antidiabetic and dilating microcirculatory vascular function may provide better cardioprotection in diabetic I/R rats when good therapeutic results cannot be achieved with the I-Post regimen alone.

### Diabetes impairs the cardioprotective effect of nicorandil on myocardial I/R injury

As previously mentioned, nicorandil attenuates I/R injury in the myocardium and reduces apoptosis in the cardiomyocytes of rats with diabetic cardiomyopathy. In addition, nicorandil, as a drug that improves microvascular circulation [27], causes vasodilation through two pathways: first, it has a nitrate-like effect, providing NO, and second, it selectively opens ATP-sensitive potassium channels (K_ATP_) in cell membranes and mitochondria [28]. Moreover, clinical studies have revealed that intracoronary injection of nicorandil prior to PCI for ST-segment elevation myocardial infarction (STEMI) significantly improved myocardial perfusion and reduced arrhythmias during PCI in patients with STEMI [29] and effectively reduced the incidence of adverse cardiovascular events. It also improves cardiac function in patients undergoing elective PCI [30]. Due to its aforementioned cardioprotective functions, we suggest that nicorandil combined with I-Post may have a better cardioprotective effect in improving diabetic myocardial I/R injury.

However, there is still a lack of studies investigating whether nicorandil can exert a protective effect in diabetic myocardial I/R injury. In our study, we applied nicorandil for the first time in diabetic I/R rats, and we found that the nitrate-like effect of nicorandil was unaffected by diabetes and had a beneficial effect on increasing plasma NO levels. However, we also found that although the area of myocardial injury was somewhat reduced in nicorandil-treated diabetic I/R rats, the degree of myocardial injury was still higher than that in nicorandil-treated nondiabetic I/R rats, which may indicate that nicorandil has some benefit in treating diabetic myocardial I/R injury; however, this protective effect is still relatively weak. We concluded through further mechanistic studies that diabetes also impairs the activation of the PI3K/Akt signalling pathway by nicorandil, which affects the therapeutic effect of nicorandil.

There is no doubt that drugs with antagonistic effects on diabetes-induced cardiomyocyte apoptosis as well as dilation of coronary arteries and improvement of microcirculation applied alone cannot provide the desired therapeutic effect in diabetic I/R rats. However, this explains the necessity of combination therapy.

### Effect of I-Post combined with nicorandil on cardioprotection in diabetic myocardial I/R injury

As mentioned earlier, the complexity and severity of diabetes combined with AMI diminish the myocardial protective effect of I-Post as well as nicorandil in myocardial I/R injury, and the existing treatment options do not provide the expected therapeutic effect. Combining I-Post with nicorandil against myocardial I/R injury in diabetic patients may be an innovative concept.

To investigate the feasibility of combination therapy, the present study was the first to apply I-Post in combination with nicorandil in I/R rats with T2DM. Our study demonstrated that the treatment regimen of I-Post combined with nicorandil was significantly superior in reducing myocardial infarct size and limiting cardiomyocyte injury compared with a single application of I-Post and nicorandil in diabetic I/R rats. The reason may be that, since both treatments have the effect of activating the PI3K/Akt signalling pathway, they can have a superimposed effect when combined and can better activate endogenous cardioprotective mechanisms. Previous studies have shown that Akt phosphorylation can activate its downstream target mToR and inhibit endoplasmic reticulum stress, thereby reducing ROS release from myocardial tissue and decreasing the degree of myocardial tissue injury [31]. Meanwhile, increased Akt phosphorylation stimulates GSK3β phosphorylation, inhibits the opening of the mitochondrial permeability translocation pore (mPTP), and reduces apoptosis in cardiac myocytes [32]. In addition, eNOS, a key target protein downstream of Akt, is a key enzyme in the induction of NO production [33], and adequate NO levels are critical for the regulation of blood flow and vasodilation [34], which play an important role in myocardial protection [35, 36]. However, our study demonstrated that these signalling pathways and key proteins, which are closely related to cardioprotective effects, did not reach ideal phosphorylation levels in I-Post- or nicorandil-treated diabetic I/R rats, which may be the reason why diabetes impairs the cardioprotective effects of I-Post and nicorandil. This resulted in the extent of myocardial damage in I-post- and nicorandil-treated diabetic I/R rats remaining severe. However, when the two treatments were combined, the degree of Akt and downstream target protein phosphorylation was significantly higher compared with that resulting from the application of I-Post or nicorandil alone. Thus, a better cardioprotective effect could be exerted.

However, the myocardial protection mechanisms differed between the two treatments. Although eNOS phosphorylation was lower in nicorandil-treated diabetic I/R rats than in nondiabetic rats, nicorandil led to not only endogenous NO production by promoting eNOS activation but also increased NO levels through the reaction of its nitrate moiety with sulfhydryl groups in vascular smooth muscle cells, resulting in no significant difference in plasma NO levels between nondiabetic and diabetic rats. When diabetes reduces the myocardial protective effect of I-Post, nicorandil can directly produce NO, reduce the aggregation and infiltration of inflammatory cells and platelets, improve coronary blood flow, and protect myocardial endothelial cells, thus protecting the heart from myocardial I/R injury. Consequently, we concluded that the therapeutic potential of existing treatment options is highly diminished by the complexity of acute myocardial infarction combined with myocardial I/R injury in diabetic patients. Therefore, I-Post combined with nicorandil against myocardial I/R injury in diabetic patients may be an innovative concept.

### Research limitations and future prospects

However, our study still has some limitations. First, many studies have shown that the opening of K_ATP_ channels is an essential mediator and end effector in the signalling pathways of I-Post [37]. As mentioned previously, nicorandil also has a role in opening K_ATP_ channels to activate cardioprotective mechanisms. However, diabetes alters the function of myocardial and vascular K_ATP_ channels, reducing the number of K_ATP_ channels in the myocardial and mitochondrial membranes; in this manner, it interferes with cardioprotection and reduces the effects of I-Post and nicorandil. Even if the upstream pathways of protection were intact during diabetes, the lack of normal function of K_ATP_ channels as terminal effectors can lead to pathway failure [12]. However, we did not investigate whether the combination of I-Post and nicorandil increased the number of protective mechanisms or the level of activation of the same mechanisms and only explored the effect of the combination treatment on the activation of the PI3K/Akt signalling pathway. We did not evaluate in depth the response curve of the most appropriate dose of nicorandil, choosing only the effective dose for clinical studies to assess its protective effect. Because the mechanism of myocardial I/R injury is very complex, our study only analysed the expression of some signalling proteins, and a series of questions regarding the signalling pathways and regulatory mechanisms related to how I-Post combined with nicorandil exerts its protective effect on diabetic myocardial I/R injury need to be further investigated.

Second, it has been demonstrated that there is no significant difference in the incidence of cardiovascular death, rehospitalization, and target lesion reimplantation between AMI patients treated with high-dose nicorandil and those treated with standard-dose nicorandil [18]; therefore, the dose of nicorandil administered in this study is the standard dose used in clinical treatment. In our study, we only confirmed that diabetes impaired the cardioprotective effect of standard-dose nicorandil in myocardial I/R injury, and it remains to be investigated whether the application of high-dose nicorandil can overcome the impairing effect of diabetes or even provide better cardioprotection in myocardial I/R injury.

Finally, although our study revealed that I-Post combined with nicorandil was more advantageous in reducing diabetic myocardial I/R injury than either treatment alone, we also found that the degree of cardiac damage was still severe compared with that in nondiabetic rats. Possible reasons for such a reduction are that apoptosis is highly involved in organ complications in diabetic patients, myocardial apoptosis in the diabetic state may lead to greater cardiac dysfunction [38, 39], and the impaired endogenous cardioprotective mechanisms induced by diabetes itself limit the therapeutic effect. Thus, the degree of myocardial damage remains higher in diabetic I/R rats than in nondiabetic I/R rats after combination treatment. At least in theory, combination therapy is a reasonable approach to limit infarct size in patients with acute myocardial infarction, but the value of combination therapy in clinical studies needs to be supported by reliable data from long-term, stable, basic studies. For example, a study of remote ischaemic preadaptation combined with exenatide for the treatment of I/R injury in porcine myocardium [40], while achieving satisfactory results in animal studies, failed to demonstrate a significant clinical benefit of the combination regimen in patients with acute myocardial infarction after translation to the COMBAT-MI trial [41]. Undoubtedly, the complexity of clinical patients often affects the results of animal studies; thus, animal models that simulate different comorbidities are more interesting to study. As previously mentioned, the complexity of diabetic comorbid AMI often affects the available therapies, and the combined application of several different cardioprotective measures to reduce myocardial I/R injury in diabetic patients has a better cardioprotective effect. Moreover, the study of diabetic myocardial I/R injury should not be limited to the cardiovascular field; an integrated, multidisciplinary approach is the future trend in the treatment of complex and severe diseases.

## Conclusion

Our study demonstrated that the severity and complexity of diabetes combined with AMI diminished the cardioprotective effect of I-Post or nicorandil alone on diabetic myocardial I/R injury. However, a regimen of I-Post combined with nicorandil may provide better protection against diabetic myocardial I/R injury by enhancing activation of the PI3K/Akt signalling pathway and increasing plasma NO levels. Therefore, we concluded that combination therapy is more advantageous for the treatment of myocardial I/R injury in combination with diabetes.

## Supplementary Information

**Additional file 1.** **Supplementary Table 1.** Diabetes modeling situation. **Supplementary Table 2.** Myocardial ischemia-reperfusion modeling situation. **Supplementary Figure 1.** Flowchart of experimental protocol. **Supplementary Figure 2.** Heart samples.

## Data Availability

The data generated and analyzed during the current study available from the Prof. Zhexun Lian on reasonable request.
