# Supplementary material for: Protective effect of ischaemic postconditioning combined with nicorandil on myocardial ischaemia‒reperfusion injury in diabetic rats
Source: BMC Cardiovasc Disord. 2022 Dec 3;22:518. doi: 10.1186/s12872-022-02967-1 (PMC9719207; doi:10.1186/s12872-022-02967-1)
Supplement: Supplementary file 1 — Additional file 1. Supplementary Table 1. Diabetes modeling situation. Supplementary Table 2. Myocardial ischemia-reperfusion modeling situation. Supplementary Figure 1. Flowchart of experimental protocol. Supplementary Figure 2. Heart samples. [file 12872_2022_2967_MOESM1_ESM.docx]

**Supplementary Table1. Diabetes modeling situation**

|  | Model completion | Model unsuccessful | Death | Survival | Total | DM model success rate | DM model failure rate |
| --- | --- | --- | --- | --- | --- | --- | --- |
| Rats number | 144 | 32 | 31 | 145 | 176 | 81.82% | 18.18% |

**Table1. Diabetes modeling situation：**A total of 176 rats were included in the diabetic group in this study. The final survivors were 145 and 31 died. Of the 145 surviving rats, 144 rats were finally confirmed to be consistent with the diabetic phenotype, with a modeling rate of about 81.82%.

**Supplementary Table2.Myocardial ischemia-reperfusion modeling situation**

|  | Model completion | Model unsuccessful | Death | Survival | Total | I/R model success rate | I/R model failure rate |
| --- | --- | --- | --- | --- | --- | --- | --- |
| MIRI modle | 60 | 26 | 17 | 69 | 86 | 69.77% | 30.23% |
| DM MIRI modle | 60 | 52 | 36 | 76 | 112 | 53.57% | 46.42% |

**Table2.Myocardial ischemia-reperfusion modeling situation:** In this study, a model of myocardial ischemia-reperfusion (I/R) injury was constructed by ligating the left anterior descending (LAD) branch. A total of 86 rats were included in the MIRI group, 17 rats died intraoperatively, 69 rats finally survived, and 60 rats were successfully modeled, with a modeling rate of about 69.77% for the I/R model. A total of 112 rats were included in the diabetic MIRI (DM MIRI) group. 36 rats died intraoperatively, and 76 rats survived, of which 60 rats were successfully modeled, with a modeling rate of about 53.57% for the DM I/R model.

**Supplementary Figure1**

**
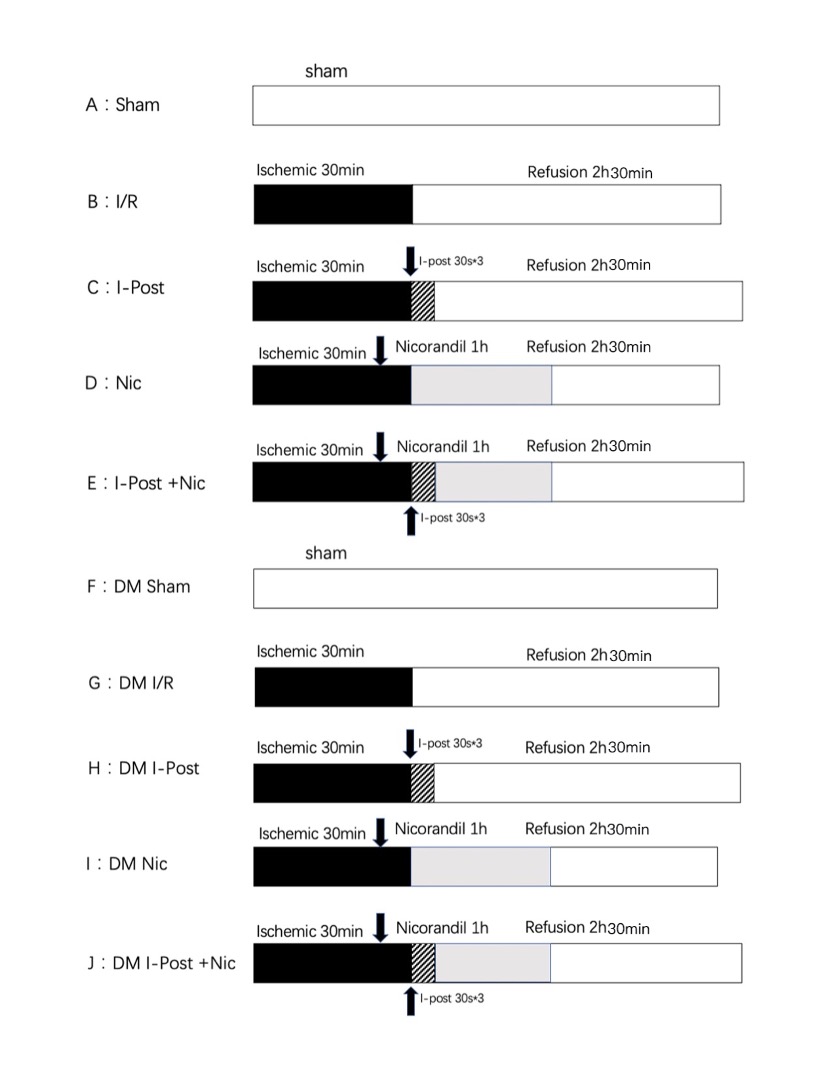
**

**Flowchart of experimental protocol:** Black block: time to left anterior descending (LAD) coronary artery occlusion. White block: time to reperfusion. Striped block: time of I-Post processing. Gray block: time of nicorandil processing. Sham, sham surgery; I/R, ischemia-reperfusion; I-Post, ischemia postconditioning; Nic, nicorandil; I-Post+Nic, ischemia postconditioning combined with nicorandil; DM, diabetes mellitus.

**Supplementary Figure2**


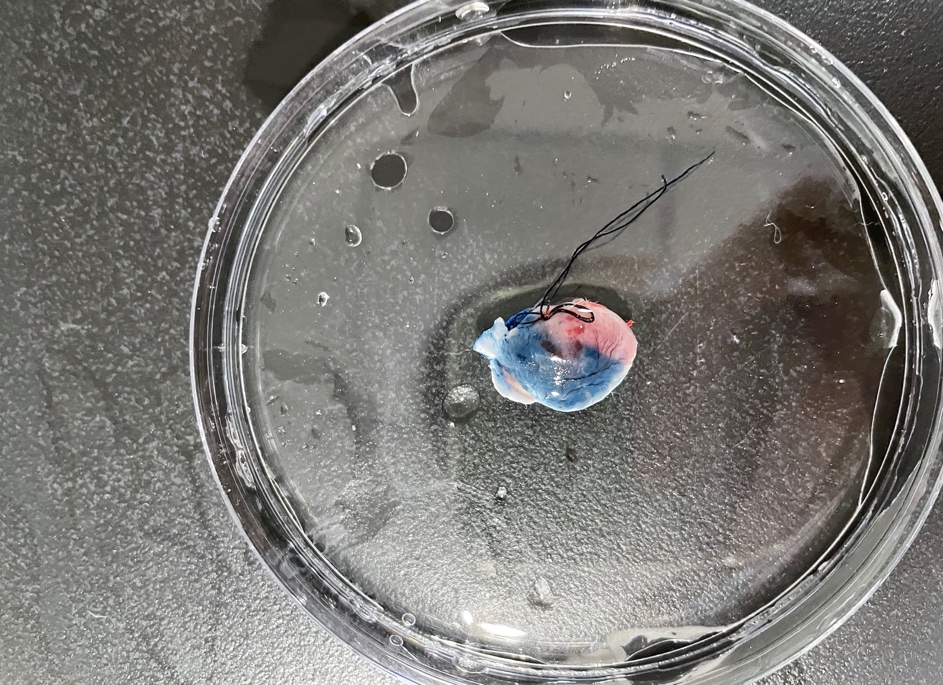


**Heart samples：**After the end of reperfusion, the rats were euthanized with a single intraperitoneal injection of an overdose of sodium pentobarbital (150 mg/kg). The LAD was then re-blocked, the left ventricle (LV) was filled with 1% Evans blue staining solution to show the ischemic risk area (AAR), and the heart samples were removed for subsequent processing.
